# Internal transcribed spacers enable species-level Metataxonomic analysis of ciliated protozoa

**DOI:** 10.1093/ismeco/ycaf024

**Published:** 2025-02-11

**Authors:** Sripoorna Somasundaram, Zhongtang Yu

**Affiliations:** Department of Animal Sciences, Animal Science Building, 2029 Fyffe Road, Columbus, OH 43210, United States; Center of Microbiome Science, 352K Wiseman Hall, 400 W 12th Ave, Columbus, OH 43210, United States; Department of Animal Sciences, Animal Science Building, 2029 Fyffe Road, Columbus, OH 43210, United States; Center of Microbiome Science, 352K Wiseman Hall, 400 W 12th Ave, Columbus, OH 43210, United States

**Keywords:** free-living ciliates, internal transcribed spacers, ribosomal RNA genes, rumen ciliates

## Abstract

Traditional morphology-based ciliate classification is often time-consuming and inaccurate, necessitating molecular approaches. Although 18S rRNA gene sequencing is widely used for taxonomic analyses of ciliates, its high degree of conservation makes it challenging to achieve species-level resolution. This study explores the potential of internal transcribed spacers (ITS1 and ITS2) and the 28S rRNA gene to improve taxonomic resolution beyond that offered by 18S rRNA gene in free-living and host-associated ciliates. A comparative analysis of ITS, the 18S, and 28S rRNA gene sequences retrieved from public databases indicated that ITS regions exhibit greater inter- and intra-specific sequence dissimilarity compared to 18S rRNA gene, supporting existing literature. We then designed universal primers targeting the ITS and 28S rRNA gene for freshwater and rumen ciliates. These primers were rigorously evaluated for their inclusiveness, specificity, and amplification efficiency using *in*-*silico* PCR, experimental PCR, followed by sequencing and metataxonomic analyses of the ciliate communities. *In*-*silico* analyses revealed inclusiveness exceeding 80%, while experimental analyses validated their specificity. Metataxonomic analyses of ciliates demonstrated that the ITS and 28S rRNA gene captured significantly greater taxonomic diversity than 18S rRNA gene. Also, ITS1 offered superior taxonomic resolution by detecting the most ciliate species that went unnoticed by the 18S rRNA gene. These findings underscore the superiority of ITS1, and to a lesser extent ITS2, as taxonomic markers for enhancing the resolution of freshwater and rumen ciliate communities. We recommend ITS1 as an alternative marker to overcome the limitations of 18S rRNA gene-based approaches in free-living and host-associated ciliate taxonomy.

## Graphical Abstract

**Graphical Abstract:**
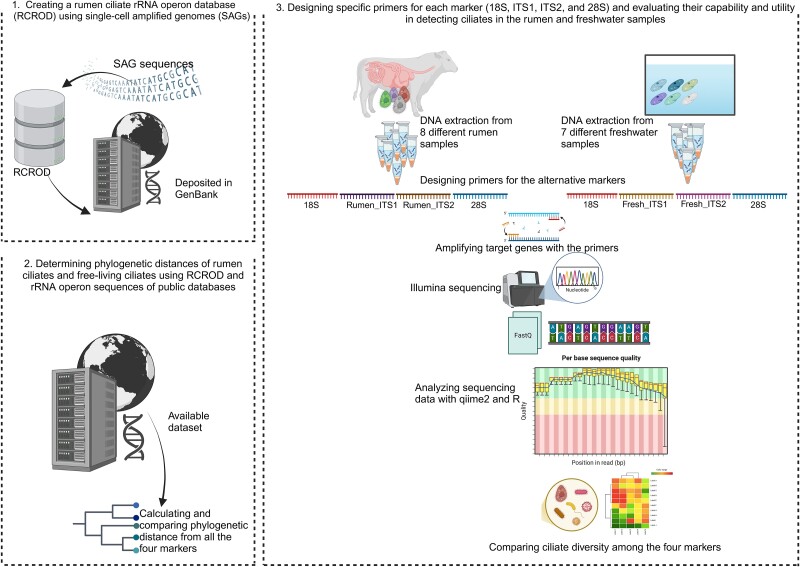

## Introduction

Microbiomes play pivotal roles in ecosystems, and significant research efforts have been directed towards microbiome studies in various ecosystems over the past two decades. However, most studies have focused primarily on bacteria, archaea, and viruses, neglecting fungi and ciliates. Ciliates are predators, and by preying on or forming symbioses with prokaryotes, ciliates regulate the population dynamics and metabolic processes of prokaryotes [1], thereby playing an integral role in shaping the microbial food webs and altering energy transfer across ecosystem trophic levels [2–4]. Ciliates can either be free-living or host-associated. Free-living ciliates are found in various environments, viz., freshwater, marine, and soil. They are diverse and classified into 12 classes within the phylum Ciliophora [5]. In contrast, host-associated ciliates live in symbiosis with their host animals in specific organs, particularly the rumen of ruminants (wild and domesticated) and other gastrointestinal regions. They are much less diverse than free-living ciliates and are classified exclusively into the class Litostomatea. Rumen ciliates are anaerobic and contribute to feed digestion, rumen pH regulation, nitrogen metabolism, and methane emissions [6]. Despite their crucial roles, ciliate diversity, population dynamics, and interactions with prokaryotes remain poorly understood. This knowledge gap has profound implications for comprehending their roles within ecosystems [7]. It is thus essential to uncover the often-overlooked diversity of ciliates and investigate their community profiles. The inclusion of ciliates in metataxonomic analysis will support holistic studies of microbiomes.

Traditionally, ciliate identification and classification were solely based on morphological features [8]. However, ciliates have relatively simple morphological features, which make it challenging to identify ciliates at the species level. Moreover, some ciliates cannot be cultured in the laboratory [9], requiring experience and expertise to distinguish their minute diagnostic features [10]. To circumvent these limitations, researchers used the small subunit ribosomal RNA (18S rRNA) gene for identification, classification, and community profiling of ciliates [11–13]. However, the highly conserved nature of the 18S rRNA gene limits its capability and utility in species-level delimitation [14, 15]. A few studies have explored alternative taxonomic markers for analyzing ciliate diversity, such as the 28S rRNA gene [16, 17], *cox*1 [10, 18, 19], *hsp*90 [20], ITS [21–24], and *rpo*B [25]. However, the primers designed for these markers are specific to only a few classes or genera of free-living ciliates. Furthermore, while genome-resolved metagenomics enables comprehensive analysis of bacteria, archaea, and viruses in various microbiomes [26–28], its application to ciliate community analyses remains challenging. This difficulty arises from the unique genomic structures of ciliates, viz., large genomes, nuclear dimorphism, high ploidy, chromosomal fragmentation [1, 29], limited numbers of sequenced ciliate genomes in databases, and their relatively low abundance in microbiomes. Consequently, metataxonomics remains the primary omics approach for profiling ciliate communities in various ecosystems.

Being intrinsically more divergent than the rRNA genes, ITS regions are widely used in mycobiome analysis for species identification and profiling [30, 31], offering significantly enhanced taxonomic resolution than the 18S rRNA gene. While ITS regions have been used in studies of free-living ciliates to infer their evolution, discover new ciliate species, and refine their phylogeny among closely related species [32–36], their potential for metataxonomic analysis of ciliate communities has yet to be explored. We hypothesize that ITS1, ITS2, and the hypervariable regions of 28S rRNA gene (hereafter all referred to as alternative markers) could provide a finer taxonomic resolution, thereby enhancing the taxonomic classification of ciliates. To test this hypothesis, we compared the 18S rRNA gene and alternative markers for sequence dissimilarity, designed primers specifically targeting each alternative marker, and evaluated their utility in the metataxonomic analysis of freshwater and rumen ciliates. Our findings demonstrate that these alternative markers, particularly ITS1, improve the taxonomic analysis of ciliate communities in freshwater and rumen environments beyond what is achievable using the 18S rRNA gene.

## Materials and methods

### PCR primer design and *in-silico* validation

**Figure 1:**
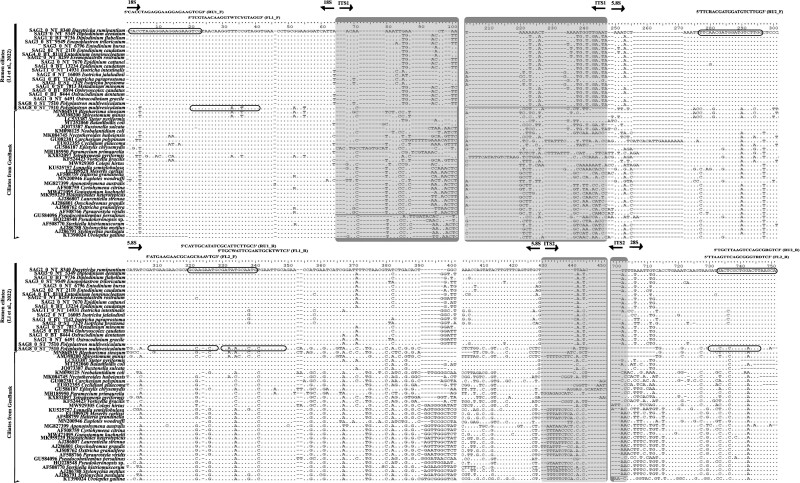
Alignment of ITS sequences of rumen and freshwater ciliates and the locations of the primers. The two sets of primers, i.e. RU1_F and RU1_R, and RU2_F and RU2_R, represent the forward and reverse primers for rumen ciliates for ITS1 and ITS2 regions, respectively. Similarly, FL1_F and RL1_R and FL2_F and RL2_R indicate the forward and reverse primers for freshwater ciliates for ITS1 and ITS2 regions, respectively. The ITS1 and ITS2 regions are shaded in grey.

**Figure 2:**
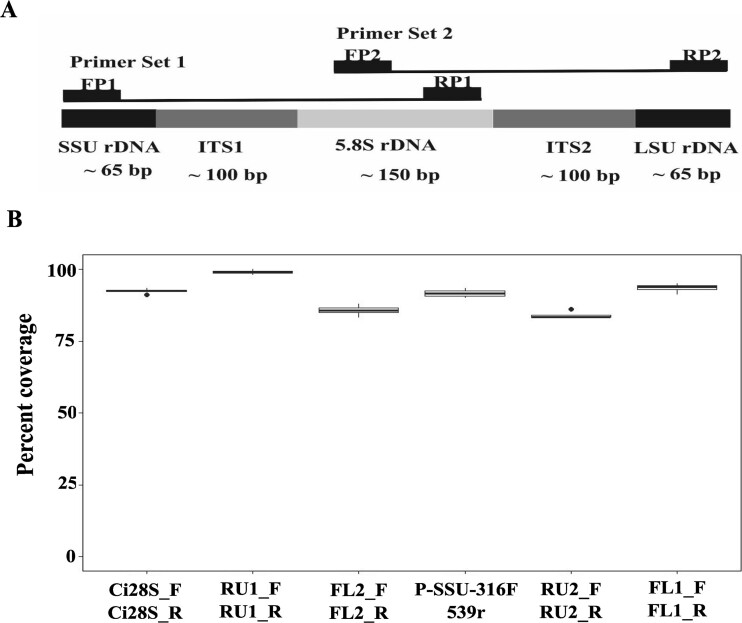
(A) A schematic showing the locations of the designed primers to amplify the complete internal transcribed spacer regions, ITS1 and ITS2, and flanking rRNA genes of ciliates. (B) Comparison of the overall performance (in percent coverage) of the different primer sets used in this study by *in*-*silico* PCR analysis. FP = forward primer, RP = reverse primer.

We conducted a systematic search of public databases to find sequences covering the ITS1 and ITS2 regions of ciliates, along with 18S, 5.8S, and 28S rRNA genes, and retrieved ~500 sequences (cultured and taxonomically assigned) for free-living ciliates from GenBank. However, our search yielded only a small number of contiguous sequences in GenBank that encompass the ITS regions and rRNA genes of rumen ciliates. We thus created an in-house rRNA operon (18S-ITS1-5.8S-ITS2-28S) database, referred to as the rumen ciliate rRNA operon database (RCROD), using the RESCRIPt plugin [37] of QIIME2-2024.2 and incorporating 52 previously reported single-cell amplified genomes (SAGs) of rumen ciliates [38]. These SAGs represent 13 genera and 19 distinct rumen ciliate species (Table S1). We combined the GenBank sequences derived from free-living and rumen ciliate sequences, aligned them using MEGA11 [39], and manually fine-tuned the alignment using BioEdit version 7.2.5 [40]. We then designed primers specific to ITS1 and ITS2 using Primer3 (https://primer3.ut.ee/) [41] based on conserved regions of the 18S, 5.8S, and 28S rRNA genes to enable amplification of complete ITS regions (Figs. 1 and 2a). The default parameters of Primer3 were used for primer designing, with primer lengths of 18-23 bp, Tm of 57–62°C, GC% of 30%–70%, and amplicon size shorter than 490 bp, a length suitable for Illumina 2 × 300 paired-end sequencing. Since it was impossible to design universal primers to amplify the ITS regions of both free-living and rumen ciliates, we designed separate primers for each group of ciliates (Fig. 1 and Table 1). To design primers specifically targeting the 28S rRNA gene, we retrieved ~800 28S rRNA gene sequences from GenBank after excluding uncultured/unclassified ciliates. After aligning the sequences, we designed a common primer set for both free-living and rumen ciliates based on conserved regions flanking the D1-D2 region. The online oligo design tools from ThermoFisher Scientific were used to calculate the GC content and melting temperatures of the designed primers. We did not design primers for the 18S rRNA gene but performed an *in-silico* evaluation of extant primers from the literature and found that the previously reported forward and reverse primers [43, 44] have the best inclusiveness and specificity (Table 1). These two primers were used to benchmark the newly designed primers specific to the alternative markers.

**Table 1:** Details of primer sets for amplification of 18S rRNA gene, ITS1, ITS2, and 28S rRNA gene.

**Primer name**	**Target │Marker**	**Sequence (5′ → 3′)**	**Length (bp)**	**GC%**	**Tm (°C)**	**Annealing Temp (°C)**	**Amplicon length (bp)**	**References**
P-SSU-316F	FLC & RC **|** 18S	GCTTTCGWTGGTAGTGTATT	20	40.00	52.50	50.00 (for RC)	482	[41]
539r		CAACTGTCTCTATKAAYCG	19	42.00	50.00	57.70 (for FLC)		[42]
RU1_F	RC **|** ITS1	CACCTAGAGGAAGGAGAAGTCG	22	54.55	61.60	55.00		Present study
RU1_R		CAYTGCATATCGCATTCTTGC	21	45.24	57.45		200	
FL1_F	FLC **|** ITS1	TCGTAACAAGGTWTCYGTAGG	21	45.24	57.68	66.00		Present study
FL1_R		TGCWATTCGCAKTGCKTWTC	20	45.00	55.23		295	
RU2_F	RC **|** ITS2	TTCRACGATGGATGTCTTGG	20	47.50	56.25	58.50		Present study
RU2_R		TGCTTAAGTCCAGCGRGTC	19	55.50	59.65		335	
FL2_F	FLC **|** ITS2	ATGAAGAACGCAGCSAAVTG	20	48.34	56.77	68.20		Present study
FL2_R		TTAAGTTCAGCGGGTRDTC	19	46.67	55.80		365	
Ci28S_F	FLC & RC **|** 28S	CGATAGCRWACAAGTACTRYGAA	23	41.24	57.19	50.00 (for RC)	271	Present study
Ci28S_R		GACTCCTTGGTYCGTGTTTC	20	53.75	58.76	62.00 (for FLC)		

**Note:** FLC = Free-living ciliates; RC = Rumen ciliates; Ci = ciliates

To assess the specificity of the designed primers, we performed *in*-*silico* PCR using Fast-PCR [42] with various amplification stringency settings, allowing for fewer than three mismatches per primer-target pairing. The primers that “amplified” more than 80% of the respective ciliate sequences (Fig. 2) were considered inclusive and synthesized for experimental evaluation.

### Assessment of the marker sequences for taxonomic resolution

We selected the sequences that span the complete rRNA operons (18S-ITS1-5.8S-ITS2-28S) to assess the taxonomic resolution of each marker. In total, 109 and 66 complete rRNA operons were obtained from free-living and rumen ciliates, respectively. The sequences of individual markers (target region of the 18S and 28S rRNA genes and full length of each ITS) were sliced out, grouped separately for free-living and rumen ciliates, and aligned using MEGA11. Following manual fine-tuning of the alignment sequences using ClustalX v2 [45], a distance matrix was generated for each marker with Tamura 3-parameter model [46].

The sequences were subjected to taxonomic classification using the QIIME2 pipeline [47]. The 18S and 28S rRNA gene sequences of free-living ciliates were classified using SILVA_138.1_SSURef_NR99 and SILVA_138.1_LSURef_NR99 databases, respectively. The ITS sequences of free-living ciliates were classified using both the NCBI ITS RefSeq and UNITE database (https://unite.ut.ee/). The NCBI ITS RefSeq database allowed more taxonomically assigned ITS sequences than UNITE database, so we chose the former for further analysis. Since the available databases contained very few sequences of the 18S rRNA gene, ITS1, ITS2, and 28S rRNA gene of rumen ciliates, we classified the sequences with the RCROD using the q2-feature-classifier plugin that implemented the scikit-learn machine-learning algorithm [48]. The sequence, taxonomy, and classifier files of the reference databases (NCBI ITS Ref and RCROD) were evaluated to determine sequence and taxonomy entropy and F-Measure scores (Table S2) using the QIIME2 RESCRIPt plugin [37]. Further analyses were performed using the QIIME2 pipeline, and the resulting taxonomy tables were converted to Phyloseq objects for analyses in R using Phyloseq packages [49]. The total number of amplicon sequence variants (ASVs) derived from each taxonomic marker was compared. The steps for creating the NCBI ITS RefSeq and RCROD databases, along with the corresponding database files, are available in a GitHub repository (https://github.com/ssomasundaram244/Ciliate-taxonomy-database).

### Evaluation of markers for their capability and utility in metataxonomic analysis of freshwater and rumen ciliates

#### Sample collection and processing

Freshwater samples were collected from seven different freshwater bodies in Columbus, Ohio, and eight different rumen samples were collected from cannulated dairy cows housed at the Waterman Agricultural and Natural Resources Laboratory of The Ohio State University. The ciliates in the freshwater samples were enriched as described previously [50] before DNA extraction. Briefly, small pieces of boiled cabbage were added to the freshwater samples in beakers and incubated at 22°C for 24 hours, followed by an additional 48-hour incubation at 22°C in Petri dishes. The ciliate growth was microscopically verified. Each sample was then centrifuged at 2000 rpm for 2 min to pellet the ciliate cells and were used for DNA extraction. For the rumen samples, each 500 ml sample was strained through four layers of cheesecloth to remove large digesta particles and mixed with an equal volume of simplex buffer, as previously outlined [51]. The mixture was maintained at 37°C for 2 hours with continuous CO_2_ sparging to facilitate the removal of small digesta particles. The rumen ciliates were also pelleted by centrifugation at 2000 rpm for 2 min. The pellets were washed once with the Simplex buffer.

#### DNA extraction and optimization of PCR conditions

Metagenomic DNA was extracted from each pelleted sample using the Qiagen DNeasy Blood and Tissue Kit (Qiagen, Inc., Germantown, MD). The quality and quantity of the DNA extracts were evaluated using a Nanodrop spectrophotometer and agarose gel (1%) electrophoresis.

We optimized the PCR conditions for each primer set using gradient PCR by adjusting annealing temperatures. The PCR reaction mix (25 μl) contained 1x buffer, 1 μM of each primer, 60 ng DNA, 0.32 mM dNTP (0.08 mM per dNTP), 1.5 mM MgCl_2_, and 0.6 U Taq DNA polymerase. The PCR thermal cycling conditions include: initial denaturation step at 95°C for 5 min; 32 cycles of denaturing at 95°C for 40 s, annealing over a range of 50–65°C (the specific temperature for each primer set is provided in Table 1) for 40 s, and extension at 72°C for 40 s; and final extension step at 72°C for 10 min.

#### Metataxonomic analyses of freshwater and rumen ciliates

We utilized metataxonomics to evaluate the capability and utility of new primers for analyzing ciliate communities relative to the 18S rRNA gene primers. Briefly, amplicon libraries were prepared individually for each metagenomic DNA sample using each primer set, each synthesized with a unique index sequence for multiplexing. These libraries were prepared following the optimized PCR conditions. After quality checking, the amplicon libraries were pooled at an equal molar ratio and paired-end (2 × 300 bp) sequenced on the MiSeq system (Illumina). The sequencing reads were demultiplexed based on the index sequences and further analyzed using the QIIME2 v 2021.4 pipeline. Briefly, the primers were trimmed off using Cutadapt, and quality plots were generated and visualized. The regions with low base-calling accuracy were identified using the base quality score (i.e. Phred33). Bases with a quality score < 20 were trimmed off from all the reads before merging the corresponding forward and reverse reads using DADA2 within QIIME2. The merged sequences were then clustered as ASVs. As previously mentioned, for the freshwater ciliates, the SILVA databases were used to taxonomically classify ASVs derived from the two rRNA genes, whereas the NCBI ITS RefSeq database was used to classify ITS1 and ITS2 ASVs. The rumen ciliate ASVs of all four markers were classified using the RCROD database.

#### Alpha diversity estimation and statistical analyses

Before estimating alpha diversity, we assessed the level of sample saturation using rarefaction analysis in R. Alpha diversity metrics (Shannon and Simpson diversity indices, richness, and evenness) were calculated using R [52]. Statistical analyses were performed using the Mann–Whitney U test (non-parametric) and one-way ANOVA, followed by a post hoc Tukey test (parametric) in R to assess whether the alternative makers could significantly improve the detection of ciliate diversity compared to the 18S rRNA gene. Data visualization was performed with the ggplot2 [53], tidyverse [54], and vegan [55] packages in R.

## Results and discussion

The ciliate diversity in various microbiomes remains insufficiently characterized, particularly at the species level, even when analyzed by sequencing their 18S rRNA gene [56]. This is particularly true for rumen ciliates, which are closely related taxa [57, 58]. To overcome this limitation, we evaluated three alternative markers with higher sequence divergence among species than the 18S rRNA gene. When amplified with the new primers designed in this study, these alternative markers produced amplicons of an appropriate length (200-480 bp) (Table 1) for ciliate metataxonomic analysis and improved species-level resolution compared to the 18S rRNA gene.

### The database sequences of the alternative markers are more divergent than the 18S rRNA gene, providing a higher taxonomic resolution

The *in-silico* analysis of database sequences revealed that the alternative markers share a much lower similarity than 18S rRNA gene among the selected 109 free-living and 66 rumen ciliate sequences (Table 2). This aligns with previous findings among prokaryotic and other eukaryotic microbes [59, 60]. Across the free-living ciliates, the sequence similarities of ITS regions and 28S rRNA gene varied from 10% to 19%, whereas that of the 18S rRNA gene reached ~64%. Similarly, the sequence similarity for ITS1 and 28S rRNA gene of rumen ciliates was also much lower than the 18S rRNA gene (Table 2). ITS1 exhibited very low sequence similarity among free-living and rumen ciliates, 18% and 12%, respectively. However, all the markers, except for ITS1, showed a higher sequence similarity among rumen ciliates when compared to the free-living ciliates (Table 2), corroborating their limited taxonomic distribution within the class Litostomatea. Compared to the rRNA genes, ITS regions experience a lower level of mutational constraint, allowing for increased sequence variability [61], potentially enhancing the taxonomic resolution of sequence-based ciliate community analysis.

**Table 2:** Sequence^a^ similarities; numbers of ASVs assigned to known genera and species; numbers of known taxa to which the ASVs could be assigned; and numbers of known species exclusively “detected” by each marker.

**Ciliate**	**Markers**	**Sequence similarity (%)**	**Sequence entropy**	**Genus-level ASVs**	**Species-level ASVs**	**Order**	**Family**	**Genus**	**Species**	**Marker-exclusive species**
Free-living	18S	64.04	2.76	57	9	1	0^c^	6	2	1
	ITS1	18.18	2.96^b^	88│^d^ 43	85│47	6│4	15│6	15│7	20│10	8│1
	ITS2	09.94	3.04^b^	93│56	78│57	6│5	10│9	14│10	16│12	5│1
	28S	18.67	2.33^b^	38	29	0	0	3	4	2
Rumen	18S	79.24	2.87	50	50	2	2	10	11	0
	ITS1	11.57	2.99^b^	64	60	2	2	10	16	3
	ITS2	54.81	2.48^b^	53	40	2	2	7	8	0
	28S	31.79	3.08^b^	3	3	1	1	1	1	0

a The sequences for free-living ciliates were downloaded from NCBI ITS RefSeq (109 sequences, each representing one ASV) that encompass the complete operons (18S-ITS1-5.8S-ITS2-28S rRNA), and the sequences for rumen protozoa (66 sequences, each also representing one ASV) were from one custom database containing 52 single-cell-amplified genomes reported by Li et al. (2022).

b The *P* value <0.001 in comparison to 18S rRNA gene.

c The lack of information in the SILVA/NCBI ITS RefSeq databases used for the current investigation could be the reason for the “0” values of orders and families detected by the 18S and 28S rRNA gene sequences.

d For free-living ciliates, the values before and after the “│” were based on the NCBI ITS RefSeq database and the UNITE database, respectively. The ITS sequences of rumen ciliates were only analyzed using the custom database.

Sequence entropy values also indicated that the ITS regions had a significantly higher entropy than 18S rRNA gene for free-living and rumen ciliates (Table 2). Given their higher entropy, ITS markers can better delimitate species than the 18S rRNA gene. Although highly conserved 18S rRNA gene markers may allow more robust global phylogeny and taxonomic assignments, highly variable ITS regions can help resolve ciliate taxonomy at lower taxonomic levels.

Sequence analysis of 109 free-living ciliates and 66 rumen ciliates among the markers revealed that the ASVs derived from the ITS regions represented a greater number of species compared to those derived from 18S and 28S genes. Additionally, a higher proportion of ITS ASVs, particularly ITS1 ASVs, were assigned to known species (Table 2). Also, more known orders, families, and genera of both free-living and rumen ciliates were identified by ASVs derived from ITS regions. Compared to the ASVs derived from the 18S rRNA gene, fewer ASVs of 28S rRNA gene were assigned to known genera or species, which is likely due to the smaller number of 28S rRNA gene sequences than 18S rRNA gene in the SILVA Ref database (24 708 vs.167330). Collectively, the sequences of the two ITS markers enhanced the taxonomic classification of ASVs at the order, family, genus, and species levels beyond that achievable by the two rRNA genes. These findings demonstrate that both ITS regions, particularly ITS1, can enhance the identification of freshwater and rumen ciliates.

Some of the genera represented by the database sequences encompassed multiple species, including closely related ones (phylogenetic distance = 0 or 0.01 based on the 18S rRNA gene). We compared the pair-wise distance matrices derived from these markers to determine if the alternative markers can differentiate the closely related species. For free-living ciliates, ITS regions were able to differentiate closely related species pairs, with the distance values ranging from 0.15 to 0.61 for ITS1 and 0.04 to 0.23 for ITS2, except for one species pair (*Holostrichides heterotypicus* and *Holostrichides obliquocirratus*), which was not adequately differentiated by ITS2 (phylogenetic distance = 0.01). In contrast, the 28S rRNA gene could not differentiate two species pairs (phylogenetic distance = 0), although the rest were differentiated with small distance matrix values (Table 3). For rumen ciliates, the ITS and 28S rRNA gene were able to distinguish all the closely related species pairs (phylogenetic distance ranging from 0.02 to 0.05) except for two species pairs and one species pair, which were not distinguished by ITS2 and 28S rRNA gene, respectively (Table 3). The highest pair-wise distance between closely related species was observed from ITS1, followed by ITS2 and the 28S rRNA gene. These findings are consistent with those reported for five astome ciliate species [24] and demonstrate that ITS1 can differentiate closely related ciliate species better than the 18S rRNA gene.

**Table 3:** Phylogenetic distance (mean ± SD) between two select species that each have multiple database sequences.

**Species**	**18S rRNA gene**	**ITS1**	**ITS2**	**28S rRNA gene**
**Free-living ciliates**				
*Holostrichides heterotypicus* vs *Holostrichides obliquocirratus*	0.00 ± 0.00^*^	0.60 ± 0.00^**a**^	0.01 ± 0.00^**^	0.00 ± 0.00^*^
*Spirostomum dharwarensis* vs *Spirostomum ambiguum*	0.01 ± 0.00^**^	0.61 ± 0.37^**b**^	0.15 ± 0.10	0.01 ± 0.00^**^
*Spirostomum dharwarensis* vs *Spirostomum minus*	0.01 ± 0.00^**^	0.57 ± 0.36^**c**^	0.08 ± 0.05	0.02 ± 0.01
*Spirostomum dharwarensis* vs *Spirostomum subtilis*	0.01 ± 0.00^**^	0.51 ± 0.49	0.15 ± 0.10	0.01 ± 0.00^**^
*Spirostomum minus* vs *Spirostomum ambiguum*	0.01 ± 0.00^**^	0.49 ± 0.45^**a**^	0.23 ± 0.05^**a**^	0.01 ± 0.00^**^
*Spirostomum minus* vs *Spirostomum subtilis*	0.01 ± 0.00^**^	0.15 ± 0.01^**a**^	0.05 ± 0.01	0.01 ± 0.01^**^
*Spirostomum teres* vs *Spirostomum teres*	0.00 ± 0.00^*^	0.44 ± 0.31^**a**^	0.04 ± 0.02	0.02 ± 0.01
*Stentor pyriformis* vs *Stentor amethystinus*	0.00 ± 0.00^*^	0.51 ± 0.21^**a**^	0.12 ± 0.06^**c**^	0.00 ± 0.00^*^
**Rumen ciliates**				
*Entodinium bursa* vs *Entodinium caudatum*	0.00 ± 0.00^*^	0.03 ± 0.02^**b**^	0.01 ± 0.00^^**^^	0.01 ± 0.00^^**^^
*Entodinium bursa* vs *Entodinium loginucleatum*	0.00 ± 0.00^*^	0.02 ± 0.01	0.03 ± 0.02	0.04 ± 0.02^**c**^
*Isotricha jalaludinii* vs *Isotricha intestinalis*	0.00 ± 0.00^*^	0.02 ± 0.01	0.00 ± 0.00^*^	0.05 ± 0.03^**b**^
*Isotricha jalaludinii* vs *Isotricha paraprostoma*	0.00 ± 0.00^*^	0.04 ± 0.02^**c**^	0.04 ± 0.01	0.04 ± 0.02^**c**^
*Isotricha prostoma* vs *Isotricha jalaludinii*	0.00 ± 0.00^*^	0.04 ± 0.03	0.04 ± 0.02	0.03 ± 0.02

**Note:** Distance was calculated with the Tamura 3-parameter model applied. 0 and 0.01 distances are represented by ^*^ and ^**^, respectively.

The superscripts indicate the level of significance when compared to the 18S rRNA gene: a, *P* < .01; b, *P* = .01; c, *P* < .05.

### The alternative markers improve metataxonomic analysis of freshwater and rumen ciliates.

#### Summary of sequencing data

The newly designed primers for the alternative markers successfully amplified their respective targets. The resulting amplicons (<490 bp) are of appropriate lengths for sequencing using the MiSeq system (Illumina, Inc.), the primary sequencing technology used for metataxonomic analyses.

We subsequently compared the four markers for their capability to identify ciliates. Following filtering, denoising, and removal of chimeric sequences, we obtained 415 688 sequences of the 18S rRNA gene, 792 360 ITS1 sequences, 308 212 ITS2 sequences, and 241 117 sequences of 28S rRNA gene from the rumen samples. The freshwater samples yielded 17 706 sequences of the 18S rRNA gene, 157 706 ITS1 sequences, 306 636 ITS2 sequences, and 93 578 sequences of 28S rRNA gene. The number of quality-checked sequences ranged from 2000 to 89 000 per sample for each marker.

#### Ciliate richness and diversity detected in freshwater and rumen samples

**Figure 3:**
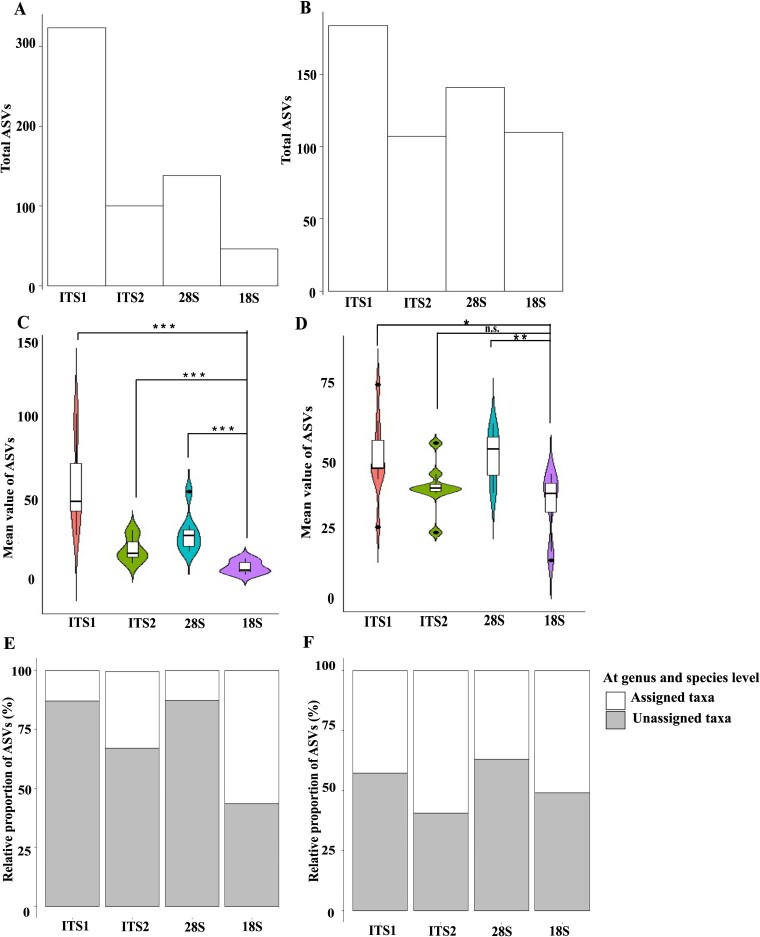
Taxonomic richness determined by the four taxonomic markers in the freshwater (A, C, E) and the rumen (B, D, F) samples. (A, B) Total number of ASVs. (C, D) mean numbers of ASVs. (E, F) percentage of assigned and unassigned taxa at the species level. Statistical significance was tested using one-way ANOVA and Mann–Whitney U test: ^***^, *P* < .01; ^**^, *P* = .01; ^*^, *P* < .05.

The alternative markers generated more ASVs from both freshwater and rumen samples, and a higher proportion of them are classified to known taxa (Fig. 3). Statistical analyses revealed that the alternative markers yielded a significantly greater number of ASVs than the 18S rRNA gene from the freshwater samples (*P* < .05). From the rumen samples, ITS1 and the 28S rRNA gene, but not ITS2, yielded more ASVs (*P* < .05) than the 18S rRNA gene.

**Figure 4:**
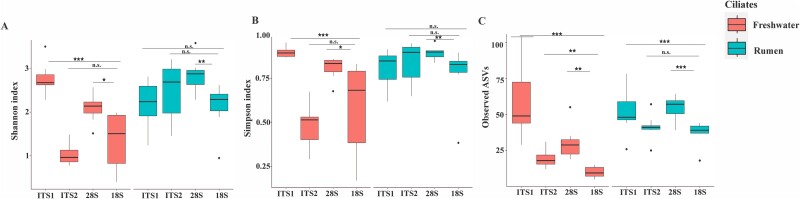
Alpha diversity indices of freshwater and rumen ciliates. Statistical significance was tested using one-way ANOVA and the Mann–Whitney U test. ^***^, *P* < .01; ^**^, *P* = .01; ^*^, *P* < .05; and n.s., not significant.

The alpha diversity analysis revealed significantly greater (*P* < .05) diversity within the freshwater ciliate communities when assessed using ITS1 and 28S rRNA gene than the 18S rRNA gene, as indicated by both Shannon diversity index and species richness (Fig. 4). This trend was also observed across the rumen ciliate communities, except for ITS1 with respect to diversity indices (Shannon and Simpson), which is non-significantly greater than the 18S rRNA gene. The greater diversity indices and species richness of rumen ciliates detected using ITSI than ITS2 align with much lower sequence similarity of the former than the latter (Table 2). Compared to the other two markers, the heightened alpha diversity captured by ITS1 and the 28S rRNA gene aligns with their elevated sequence variability. This result aligns with an earlier study showing that the D1-D2 hypervariable region of 28S rRNA gene in ciliates exhibits a higher variability and, thus, greater power to distinguish ciliate species than the 18S rRNA gene [56]. Similarly, the D2 hypervariable region of the fungal 28S rRNA gene could capture a higher level of diversity than ITS2 [62].

#### Ciliate species and higher taxa detected and identified

We compared the percentages of ASVs classified to known taxa (from class to species) among the taxonomic markers. In the freshwater samples, only ~10% of the ASVs detected by the 28S rRNA gene were classified to a known taxa, whereas a higher percentage of ASVs detected by the other three markers were classified (Fig. 3E, F). In the rumen samples, the percentages of assigned and unassigned ASVs were similar across all four taxonomic markers, except for ITS1, which showed a relatively lower percentage of assigned ASVs. Despite the lower percentage of classified ASVs derived from ITS1 in the rumen samples and the 28S rRNA gene in the freshwater samples, these two markers detected a greater number of total ASVs, highlighting their potential for identifying novel or uncharacterized taxa. This contrast underscores the trade-off between taxonomic resolution and the ability to detect diverse ASVs, depending on the marker and sample type.

Within the freshwater samples, ITS1, followed by ITS2, detected more classes, orders, families, genera, and species than the two rRNA genes (Table 4). The rRNA genes did not detect any orders or families, except for one order detected by the 18S rRNA gene. This poor detection of classified taxa of freshwater ciliates at these taxonomic ranks can be attributed to the limited representation of classified ciliates in the SILVA database, compounded by potential PCR bias. To validate this premise, we searched the SILVA database for the classes, orders, and families of free-living ciliates recorded in the NCBI ITS RefSeq database. Although the SILVA_138.1_SSURef_NR99 and SILVA_138.1_LSURef_NR99 databases contain some subclasses and suborders of protozoa, each contains a smaller number of classes, orders, and families as the NCBI ITS RefSeq database: 11 classes, nine orders, and two families of ciliates were recorded in SILVA_138.1_SSURef_NR99, and only four classes and one family were recorded in SILVA_138.1_LSURef_NR99. In contrast, the NCBI ITS RefSeq database was significantly more comprehensive, documenting 11 classes, 34 orders, and 108 families. A further comparison revealed that SILVA_138.1_SSURef_NR99 and NCBI ITS RefSeq had the same set of 11 ciliate classes (*Litostomatea*, *Armophorea*, *Plagiopylea*, *Oligohymenophorea*, *Phyllopharyngea*, *Spirotrichea*, *Nassphorea*, *Karyorelictea*, *Colpodea*, *Prostomatea*, and *Heterotrichea*), whereas SILVA_138.1_LSURef_NR99 contained only four of the classes (*Litostomatea*, *Prostomatea*, *Oligohymenophorea*, and *Spirotrichea*). Furthermore, only four orders were shared between SILVA_138.1_SSURef_NR99 and NCBI ITS RefSeq. At the family level, only *Oxytrichidae* and *Mesodiniidae* were found in both SILVA_138.1_SSURef_NR99 and NCBI ITS RefSeq, and *Mesodiniidae* is the only family documented across all three databases. These findings indicate that the NCBI ITS RefSeq database has more classified taxa and a better-defined ciliate taxonomy than the two SILVA databases, which can improve the metataxonomic analysis of free-living ciliates.

**Table 4:** Numbers of ASVs | number taxa assigned at different taxonomic levels; and species evenness detected by each phylogenetic marker.

**Markers**	**Class**	**Order**	**Family**	**Genus**	**Species**	**Pielou’s evenness (mean** ± **S.D.**)
	**ASVs | Taxa**	**ASVs | Taxa**	**ASVs | Taxa**	**ASVs | Taxa**	**ASVs | Taxa**	
**Free-living**						
18S	45 | 4	1 | 1	0 | 0	33 | 15	19 | 13	0.57 ± 0.23
ITS1	154 | 8	166 | 14	172 | 22	153 | 26	135 | 29	0.70 ± 0.06
ITS2	93 | 5	52 | 11	51 | 18	49 | 27	38 | 20	0.35 ± 0.08
28S	22 | 2	0 | 0	0 | 0	15 | 7	20 | 4	0.62 ± 0.09
**Rumen**						
18S	110 | 1	110 | 2	110 | 2	73 | 8	46 | 9	0.58 ± 0.13
ITS1	184 | 1	149 | 2	151 | 2	98 | 7	59 | 10	0.56 ± 0.11^a^
ITS2	107 | 1	103 | 2	103 | 2	78 | 7	49 | 8	0.67 ± 0.17^a^
28S	142 | 1	142 | 2	142 | 2	83 | 6	64 | 10	0.71 ± 0.08^a^

a signifies significance at a *P* value of <0.01 when compared to the 18S rRNA gene.

**Figure 5:**
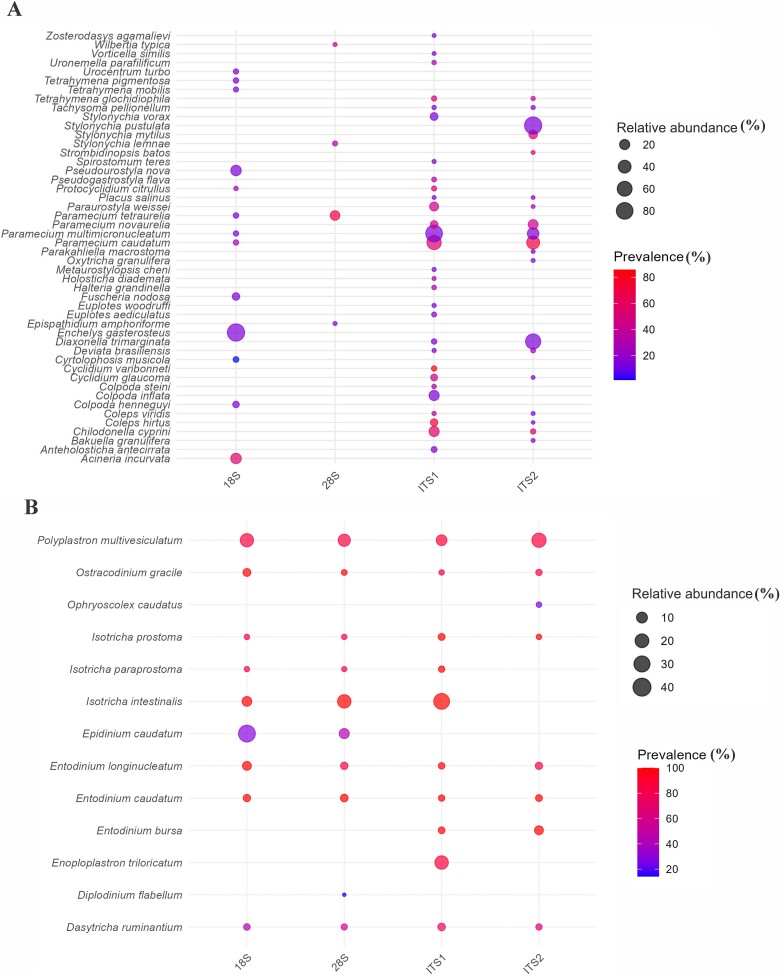
Relative abundance (%) and prevalence (%) of the identified ciliate species detected by the four taxonomic markers from (A) freshwater samples; and (B) rumen samples.

In the freshwater samples, ITS1 and the 18S rRNA gene identified 29, and 13 ciliate species, respectively. Additionally, 26 species detected by ITS1 and 17 of the ITS2 detected 20 ciliate species were not identified by 18S rRNA gene (Tables 4, S3). However, the ITS markers missed 10 species detected by the 18S rRNA gene (Fig. 5A; Table S3). The 28S rRNA gene detected only four species. These differences can partly be attributed to the limited representation of ciliate species in the current SILVA database. Despite these discrepancies, ITS1 and ITS2 together detected 13 shared species, with 16 unique to ITS1 and seven to ITS2 (Fig. 5A; Table S3). Thus, even though the two ITS markers could detect some of the freshwater ciliates, a few may escape detection by them.

In the rumen samples, ITS1 and the 28S rRNA gene outperformed 18S rRNA gene, detecting more ASVs and one additional species. The RCROD database, representing 17 species across 12 genera, helped identify 13 species from nine genera in dairy cow samples (Fig. 5B; Table S3). Although only a subset of species was detected by all markers, the alternative markers identified all species detected by the 18S rRNA gene, demonstrating their superior sensitivity for ciliate detection. These findings highlight the effectiveness of ITS1 and 28S rRNA as alternative markers, offering a more robust and comprehensive approach to characterizing rumen ciliate diversity.

**Figure 6:**
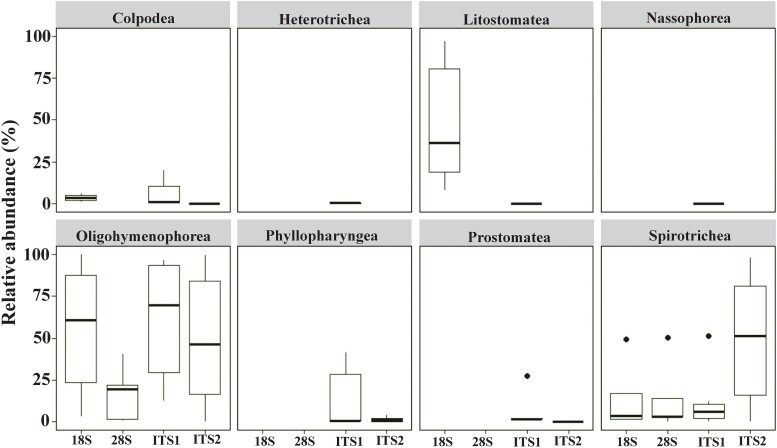
Relative abundance of classes detected by the four taxonomic markers in freshwater samples.

In the rumen samples, consistent with the previous findings, only the class *Litostomatea* was detected (Fig. 6). In freshwater samples, the majority of the 47 freshwater ciliate species detected by the four markers belong to *Oligohymenophorea* and *Spirotrichea*, while some ciliate species belonging to the classes *Heterotrichea*, *Phyllopharyngea*, and *Prostomatea* were identified only by the ITS regions (Table S3, Fig. 6). These findings underscore the complementary nature of different markers and suggest that combining multiple markers, particularly ITS1 and the 18S rRNA gene, may be used together to enhance comprehensiveness and accuracy of metataxonomic analyses of ciliate communities in freshwater ecosystems.

#### Predominance and prevalence of ciliate classes and species detected in the freshwater and rumen samples

We computed the relative abundance of individual ciliate taxa as a measure of predominance at the class level, a taxonomic rank at which morphological features serve as useful criteria for classification. Eight ciliate classes were detected in freshwater samples (Fig. S1), with their predominance varying considerably depending on the marker used. *Heterotrichea* and *Nassophorea* were detected only by ITS1, while *Phyllopharyngea* and *Prostomatea* were detected by ITS1 and ITS2. Notably, *Oligohymenophorea* and *Spirotrichea* were detected by all the markers, with ITS2 detecting both at a higher predominance compared to the other markers. Within these classes, species belonging to *Oligohymenophorea* and *Spirotrichea*, including *Paramecium caudatum, P. multimicronucleatum, Protocyclidium citrillus, Tetrahymena glochidiophila, Strombidinopsis batos, Stylonychia mytilus,* and *Stylonychia pustulata*, were detected as predominant by ITS regions (Fig. 5A; Table S3), reflecting the well-documented high prevalence of these two ciliate classes in freshwater ecosystems [63–67]. These results indicate that both ITS markers can support metataxonomic analysis of ciliate classes in freshwater ecosystems.

At the species level, the predominance of freshwater ciliates detected by the four markers also varied considerably (Table S3). While the 18S rRNA gene detected *Enchelys gasterosteus* as predominant, ITS1 and ITS2 identified *Paramecium micromacronucleatum* and *Stylonychia pustulata*, respectively, as predominant species. These discrepancies in the species detected and their predominance among the markers may result from PCR biases influenced by factors such as GC content of the markers [68], primer annealing, and amplification efficiency [69]. It is important to recognize that metataxonomic analyses only provide relative abundance data of individual microbial groups, and qPCR is needed to quantify the absolute abundance of individual ciliate taxa. The primer sets designed in this study can be used to quantify the abundance of free-living ciliates. These findings highlight the importance of marker selection in metataxonomic studies and underscore the utility of combining multiple approaches, viz., ITS-based metataxonomic analyses and qPCR, to comprehensively understand ciliate diversity and abundance.

The alternative markers consistently detected a diverse array of ciliate species in the rumen samples (Fig. 5A; Table S3), in which *Entodinium caudatum*, the most prevalent species of rumen ciliates, was detected by all four taxonomic markers. Some other species, viz., *E. bursa, E. longinuclatum, Isotricha intestinalis, I. paraprostoma,* and *I. prostoma* were also detected in most of the rumen samples, demonstrating the robustness of these markers. However, each marker identified different predominant rumen ciliates: ITS1 and the 28S rRNA gene identified *Isotricha intestinalis*, 18S rRNA gene and ITS2 identified *Epidinium caudatum*, and *Polyplastron multivesiculatum,* respectively, as the most predominant species (Table S3). More SAGs (>100) of rumen ciliates are being sequenced (personal communications, Zongjun Li, Northwest Agricultural and Forestry University, Yangling, China), and the RCROD database will be expanded to include marker sequences from these new SAGs to further enhance the metataxonomic analysis of the rumen ciliate communities. These efforts will provide a more comprehensive framework for analyzing the diversity and ecological roles of ciliate communities in the rumen microbiome.

Pielou’s evenness was calculated to assess ciliate species distribution across the markers. In freshwater ciliates, ITS1 and the 28S rRNA gene exhibited higher evenness than the 18S rRNA gene and ITS2 (Table 4). In rumen ciliates, the 28S rRNA gene and ITS2 revealed higher evenness, while ITS1 showed lower evenness than 18S rRNA gene (Table 4). These differences between freshwater and rumen ciliates suggest environment-specific differences in species detection and dominance influenced by the choice of markers. These findings underscore the importance of selecting appropriate markers for accurately assessing ciliate species evenness in diverse ecosystems.

The success of microbial species detection through metataxonomics depends on multiple factors: marker resolution, primer inclusiveness, PCR bias, and database completeness. One major limitation is the inability to detect specific taxa due to the absence of corresponding marker sequences in the database used for taxonomic classification. Additionally, database comprehensiveness affects the predominance and evenness of the detected ciliate taxa. While an artificial mock community with known species composition would provide a valuable benchmark for comparing the markers, such a resource is currently unavailable for free-living or rumen ciliates. In this study, however, our evaluations and comparisons of the markers demonstrate that ITS1, followed by ITS2, outperforms the 18S rRNA gene by resolving more ASVs and identifying more species in both the ciliate communities (Tables 4 and S3). These findings align with previous studies on fungal metataxonomics [70–72] and highlight the potential of ITS regions to enhance the resolution and accuracy of ciliate metataxonomic analyses. Future research should focus on developing standardized mock ciliate communities and refining databases to further improve the accuracy and reliability of ciliate metataxonomic studies.

In conclusion, metataxonomics remains essential for comprehensively analyzing ciliate communities across diverse ecosystems. This study evaluated four taxonomic markers (ITS1, ITS2, the 18S, and 28S rRNA genes) for their sequence similarity and taxonomic resolution, designed and verified primers specific to each marker, and assessed their capability in metataxonomic analysis of freshwater and rumen ciliates. Among these markers, ITS1 demonstrated the highest taxonomic resolution, enabling the identification of a broader range of taxa, from class to species in both ecosystems. ITS2 also showed strong performance but was less effective than ITS1, while the 18S and 28S rRNA genes provided lower resolution. Comparative analysis of databases revealed that the NCBI ITS RefSeq database, with its broader range of classified taxa, outperformed SILVA database in metataxonomic analysis of free-living ciliates. However, ITS1 failed to detect a small number of freshwater ciliates detected by the 18S rRNA gene, highlighting the need for further expansion of the NCBI ITS RefSeq database. As this database continues to expand, the utility of ITS1 in achieving comprehensive and accurate metataxonomic analysis of free-living ciliate communities across microbiomes is expected to improve. Similarly, continued expansion of the RCROD database will enhance metataxonomic studies of the rumen ciliate communities. These findings underscore the potential of ITS1 as a gold standard marker for advancing the understanding of ciliate biodiversity in diverse ecosystems.

## Supplementary Material

Figure_S1_ycaf024

Table_S1_ycaf024

Table_S2_ycaf024

Table_S3_ycaf024(1)

## Data Availability

The rumen ciliate rRNA operon database (RCROD) for the 18S rRNA gene, ITS1-5.8S-ITS2, and the 28S rRNA genes are deposited in NCBI GenBank (GenBank submission number: PP067594 to PP067647). Besides, the database (RCROD) is available in GitHub repository at https://github.com/ssomasundaram244/Ciliate-taxonomy-database.
