# Supplementary figures and images for: Internal transcribed spacers enable species-level Metataxonomic analysis of ciliated protozoa
Source: ISME Commun. 2025 Feb 11;5(1):ycaf024. doi: 10.1093/ismeco/ycaf024 (PMC11879186; doi:10.1093/ismeco/ycaf024)

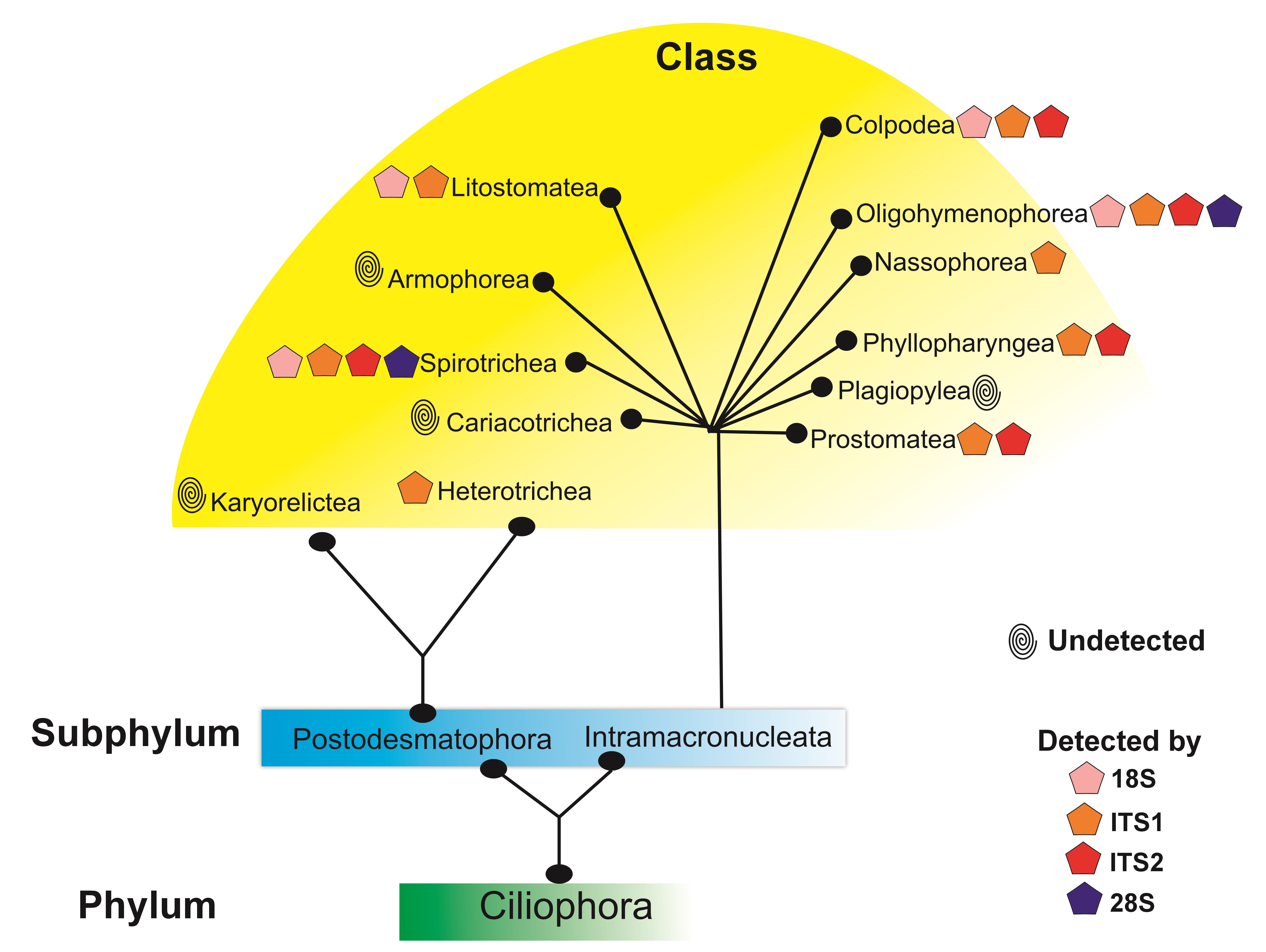

Supplement: Figure_S1_ycaf024 [file figure_s1_ycaf024.jpeg]
