# Supplementary material for: Internal transcribed spacers enable species-level Metataxonomic analysis of ciliated protozoa
Source: ISME Commun. 2025 Feb 11;5(1):ycaf024. doi: 10.1093/ismeco/ycaf024 (PMC11879186; doi:10.1093/ismeco/ycaf024)
Supplement: Table_S2_ycaf024 [file table_s2_ycaf024.docx]

**Table S2** Evaluation of sequences and taxonomies of databases created for this study.

| **Database** | **Sequence entropy** | **Taxonomic level** | **Taxonomy entropy Reference \| Predicted** | **F-Measure of classifier** |
| --- | --- | --- | --- | --- |
| NCBI ITS Ref | 7.99 | Kingdom | 0 \| 0 | 1 |
|  |  | Phylum | 0 \| 0 | 1 |
|  |  | Class | 1.36 \| 1.36 | 1 |
|  |  | Order | 2.61 \| 2.61 | 0.99 |
|  |  | Family | 3.21 \| 3.27 | 0.96 |
|  |  | Genus | 3.74 \| 3.82 | 0.94 |
|  |  | Species | 4.82 \| 4.96 | 0.85 |
| 18S rRNA gene | 3.19 | Kingdom | 0 \| 0 | 1 |
|  |  | Phylum | 0 \| 0 | 1 |
|  |  | Class | 0 \| 0 | 1 |
|  |  | Order | 0.59 \| 0.59 | 1 |
|  |  | Family | 0.59 \| 0.59 | 1 |
|  |  | Genus | 2.12 \| 2.12 | 1 |
|  |  | Species | 2.63 \| 2.63 | 1 |
| ITS1-5.8S-ITS2 | 4.72 | Kingdom | 0 \| 0 | 1 |
|  |  | Phylum | 0 \| 0 | 1 |
|  |  | Class | 0 \| 0 | 1 |
|  |  | Order | 0.57 \| 0.57 | 1 |
|  |  | Family | 0.57 \| 0.57 | 1 |
|  |  | Genus | 2.23 \| 2.23 | 1 |
|  |  | Species | 2.76 \| 2.70 | 0.95 |
| 28S rRNA gene | 3.53 | Kingdom | 0 \| 0 | 1 |
|  |  | Phylum | 0 \| 0 | 1 |
|  |  | Class | 0 \| 0 | 1 |
|  |  | Order | 0.59 \| 0.59 | 1 |
|  |  | Family | 0.59 \| 0.59 | 1 |
|  |  | Genus | 2.12 \| 2.12 | 1 |
|  |  | Species | 2.63 \| 2.63 | 1 |
