# Supplementary material for: Internal transcribed spacers enable species-level Metataxonomic analysis of ciliated protozoa
Source: ISME Commun. 2025 Feb 11;5(1):ycaf024. doi: 10.1093/ismeco/ycaf024 (PMC11879186; doi:10.1093/ismeco/ycaf024)
Supplement: Table_S3_ycaf024(1) [file table_s3_ycaf024(1).docx]

**Table S3** Relative abundance (% ± SD)│prevalence (%) of the identified ciliate species (listed per class) detected by the four phylogenetic markers.

| **Species** | **18S rRNA gene** | **ITS1** | **ITS2** | **28S rRNA gene** |
| --- | --- | --- | --- | --- |
| **Freshwater ciliates** | | | | |
| **Colpodea** | | | | |
| *Colpoda henneguyi* | 4.04±0│14.29 | NA | NA | NA |
| *Colpoda inflata* | ND | 20±0│14.29 | ND | ND |
| *Colpoda steini* | ND | 0.41±0.52│28.57 | ND | NA |
| *Cyrtolophosis musicola* | 1.97±1.41│42.86 | NA | NA | NA |
| **Heterotrichea** | | | | |
| *Spirostomum teres* | ND | 0.17±0│14.29 | ND | ND |
| **Litostomatea** | | | | |
| *Acineria incurvata* | 22.08±12.68│57.14 | NA | NA | NA |
| *Enchelys gasterosteus* | 87.61±0│14.29 | ND | ND | ND |
| *Epispathidium amphoriforme* | ND | ND | ND | 0.09±0│14.29 |
| **Phyllopharyngea** | | | | |
| *Chilodonella cyprini* | NA | 19.93±22.47│57.14 | 1.38±1.79│57.14 | NA |
| **Oligohymenophorea** | | | | |
| *Cyclidium glaucoma* | GD | 4.69±3.71│42.86 | 0.04±0│14.29 | ND |
| *Cyclidium varibonneti* | GD | 1.46±1.04│85.71 | ND | ND |
| *Fuscheria nodosa* | 6.97±0│14.29 | ND | ND | ND |
| *Paramecium caudatum* | 1.53±1.71│28.57 | 53.92±30.67│57.14 | 40.77±36.70│71.43 | ND |
| *Paramecium multimicronucleatum* | 1.37±0│14.29 | 80.27±0│14.29 | 29.46±0│14.29 | ND |
| *Paramecium novaurelia* | ND | 7.45±3.36│42.86 | 17.91±14.80│42.86 | ND |
| *Paramecium tetraurelia* | 1.76±0│14.29 | ND | ND | 16.67±16.42│71.43 |
| *Protocyclidium citrullus* | 0.34±0.18│28.57 | 1.07±1.37│57.14 | ND | ND |
| *Tetrahymena glochidiophila* | GD | 1.23±2.09│57.14 | 0.24±0.33│42.86 | NA |
| *Tetrahymena mobilis* | 1.11±0│14.29 | GD | GD | NA |
| *Tetrahymena pigmentosa* | 1.66±0│14.29 | GD | GD | ND |
| *Urocentrum turbo* | 1.33±0│14.29 | GD | 3.45±0│14.29 | ND |
| *Uronemella parafilificum* | ND | 0.35±0.31│28.57 | ND | ND |
| *Vorticella similis* | ND | 0.05±0│14.29 | ND | NA |
| *Wilbertia typica* | ND | ND | ND | 0.12±0.09│42.86 |
| **Nassophorea** | | | | |
| *Zosterodasys agamalievi* | ND | 0.05±0│14.29 | ND | ND |
| **Prostomatea** | | | | |
| *Coleps hirtus* | ND | 6.33±11.76│71.43 | 0.01±0│14.29 | ND |
| *Coleps viridis* | NA | 0.24±0.08│42.86 | 0.07±0│14.29 | NA |
| *Placus salinus* | ND | 0.05±0│14.29 | 0.02±0│14.29 | ND |
| **Spirotrichea** | | | | |
| *Anteholosticha antecirrata* | ND | 2.40±0│14.29 | ND | NA |
| *Bakuella granulifera* | ND | ND | 0.07±0│14.29 | ND |
| *Deviata brasiliensis* | NA | 0.18±0│14.29 | 0.68±0.88│28.57 | NA |
| *Diaxonella trimarginata* | ND | 1.56±0│14.29 | 63.05±0│14.29 | ND |
| *Euplotes aediculatus* | ND | 0.83±0│14.29 | ND | ND |
| *Euplotes woodruffi* | ND | 0.18±0│14.29 | ND | NA |
| *Halteria grandinella* | ND | 0.49±0.08│28.57 | ND | ND |
| *Holosticha diademata* | ND | 0.13± 0.08│28.57 | ND | ND |
| *Metaurostylopsis cheni* | GD | 0.16±0│14.29 | ND | ND |
| *Oxytricha granulifera* | ND | ND | 0.09±0│14.29 | ND |
| *Parakahliella macrostoma* | NA | ND | 0.07±0│14.29 | NA |
| *Paraurostyla weissei* | GD | 13.49±22.74│42.86 | 0.04±0.06│28.57 | GD |
| *Pseudogastrostyla flava* | ND | 0.72±0.67│28.57 | ND | NA |
| *Pseudourostyla nova* | 22.03±0│14.29 | NA | NA | NA |
| *Strombidinopsis batos* | ND | ND | 0.08±0.06│57.14 | ND |
| *Stylonychia lemnae* | ND | ND | GD | 0.93±1.09│28.57 |
| *Stylonychia mytilus* | ND | ND | 11.18±15.13│57.14 | ND |
| *Stylonychia pustulata* | ND | ND | 86.81±0│14.29 | ND |
| *Stylonychia vorax* | NA | 8.03±0│14.29 | GD | NA |
| *Tachysoma pellionellum* | ND | 0.26±0│14.29 | 0.21±0│14.29 | ND |
| **Rumen** **ciliates** | | | | |
| **Litostomatea** | | | | |
| *Dasytricha ruminantium* | 2.29±1.20│42.86 | 3.93±3.94│75 | 2±1.58│62.50 | 1.87±1.33│57.14 |
| *Diplodnium flabellum* | GD | ND | ND | 0.17±0│14.29 |
| *Enoploplastron triloricatum* | ND | 22.58±17.49│87.50 | ND | ND |
| *Entodinium bursa* | GD | 2.52±1.95│100 | 6.69±6.48│100 | GD |
| *Entodinium caudatum* | 3.53±2.93│100 | 1.99±1.97│100 | 2.65±2.21│100 | 4.04±3.04│100 |
| *Entodinium longinucleatum* | 6.64±10.54│100 | 2.24±3.45│100 | 3.48±5.20│87.5 | 3.6±4.54│85.71 |
| *Epidinium caudatum* | 40.75±7.14│28.57 | GD | GD | 10.08±8.75│42.86 |
| *Isotricha intestinalis* | 8.96±10.68│100 | 34.50±30.09│100 | GD | 22.42±30.75│100 |
| *Isotricha paraprostoma* | 0.96±0.69│85.71 | 2.06±1.66│100 | GD | 0.81±0.56│85.71 |
| *Isotricha prostoma* | 1.03±0.69│85.71 | 2.75±2.59│100 | 1.01±0.77│100 | 1.01±0.59│85.71 |
| *Ophryoscolex caudatus* | ND | ND | 1.08±0.82│25 | ND |
| *Ostracodinium gracile* | 4.44±5.01│100 | 0.98±0.97│87.50 | 1.98±1.98│87.50 | 1.34±1.17│100 |
| *Polyplastron multivesiculatum* | 21.94±30.11│85.71 | 12.08±18.73│87.50 | 26.36±28.70│87.50 | 17.53±23.26│85.71 |

Note: NA, no sequence available in the SILVA or NCBI ITS RefSeq databases representing the respective species; ND, not detected by the respective marker; GD, the species was not detected, but the corresponding genus was detected.
